# Daytime Neurophysiological Hyperarousal in Chronic Insomnia: A Study of qEEG

**DOI:** 10.3390/jcm9113425

**Published:** 2020-10-26

**Authors:** Da Young Oh, Su Mi Park, Sung Won Choi

**Affiliations:** 1 Department of Psychology, Duksung Women’s University, Seoul 01369, Korea; ponytail91@duksung.ac.kr; 2 Department of Psychiatry, SMG-SNU Boramae Medical Cneter, Seoul 07061, Korea; lovessum12@snu.ac.kr; 3 Department of Clinical Medical Sciences, Seoul National University College of Medicine, Seoul 08826, Korea

**Keywords:** chronic insomnia, hyperarousal, qEEG, ECG

## Abstract

Background: The hyperarousal model demonstrates that instability of sleep-wake regulation leads to insomnia symptoms and various neurophysiological hyperarousal states. Previous studies have shown that hyperarousal states that appear in chronic insomnia patients are not limited to sleep at nighttime but are stable characteristics that extend into the daytime. However, this phenomenon is mainly measured at bedtime, so it hard to determine whether it is maintained throughout a 24 h cycle or if it just appears at bedtime. Methods: We examined the resting state qEEG (quantitative electroencephalogram) and ECG (electrocardiogram) of chronic insomnia patients (*n* = 24) compared to good sleepers (*n* = 22) during the daytime. Results: As compared with controls, participants with insomnia showed a clearly high beta band activity in eyes closed condition at all brain areas. They showed a low frequency band at the frontal area; high frequency bands at the central and parietal areas were found in eyes open condition. Significantly higher heart rates were also found in the chronic insomnia group. Conclusion: These findings suggest that chronic insomnia patients were in a state of neurophysiological hyperarousal during the middle of the day due to abnormal arousal regulation.

## 1. Introduction

In the Diagnostic and Statistical Manual of Mental Disorders, Fifth Edition (DSM-5) [1,2], insomnia is defined as a predominant complaint of dissatisfaction with sleep quantity or quality associated with difficulty in initiating sleep (i.e., difficulty in falling asleep), difficulty in maintaining sleep (i.e., frequently or continuously awakening during the night), early morning sleep termination (awakening earlier than scheduled in the morning and difficulty falling asleep again), and fatigue despite sleeping for a sufficient amount of time [2,3]. In general, insomnia is divided into acute or short-term sleep disorders with a duration of less than three months and chronic sleep disorders with more than three months. Acute insomnia can be reversed when the initial triggering factors such as stress and anxiety disappear; however, patients with chronic insomnia complain of persistent sleep problems despite the absence of stressful events [4]. In addition, maladaptive behaviors and improper sleep habits, such as lying on the bed for an extended period without falling asleep [5,6], are risk factors that can maintain chronic insomnia. 

However, chronic insomnia can occur without perpetuating factors and the factors themselves do not cause chronic insomnia [7]. This suggests that acute insomnia and chronic insomnia are heterogeneous. Unlike acute insomnia, which occurs temporarily due to external factors, chronic insomnia may be maintained by characteristics of an individual even in the absence of external factors. In this regard, Riemann et al. [8] suggest that the internal causes and pathophysiological characteristics of chronic insomnia may be explained by the ‘Hyperarousal model’. The hyperarousal model states that insomnia is due to an unstable state, such as weak activation of the ventrolateral preoptic nucleus (VLPO), which activates sleep via GABAergic/galinergic cells, and hyperactivation of the ascending reticular activating system (ARAS), which leads to arousal via orexinergic/hypocretinergic cells. The model of neurophysiological hyperarousal in chronic insomnia has been supported by empirical evidence. Numerous studies found that hyperarousal characteristics of insomnia, such as heart rate, body temperature, metabolic rate, adrenocorticotrophic hormone (ACTH) and cortisol secretion, global cerebral glucose metabolism, frontalis and mentalis electromyographic activity, and basal skin resistance, were higher than those of normal sleepers [9,10,11,12,13,14,15,16]. In addition, electroencephalogram (EEG) studies of patients with insomnia revealed significant increases of power in the high-frequency spectrum in the insomnia group compared with the control group during the sleep onset period, nonrapid eye movement (NREM) sleep, and rapid eye movement (REM) sleep [17,18,19,20,21,22,23,24]. Moreover, despite the low total sleep time during the night, patients with insomnia also have a long sleep latency during the day [25,26,27,28] and the high-frequency power is greater in the waking period [29,30]. Previous studies suggest that the hyperarousal state observed in patients with chronic insomnia is not limited to sleep during the night but extended to the daytime, which is ‘a 24 h hyperarousal state’. Therefore, insomnia is not only confined to the inability to sleep well at night but is also associated with internal pathological traits. However, to the best of our knowledge, previous EEG studies, which identified the ‘24-h hyperarousal state’ of the cerebral cortex during waking state in chronic insomnia, were mainly conducted at bedtime, and some studies were conducted during the period prior to falling asleep or immediately after sleep onset. Some other studies were conducted during the waking state in the late evening hours when melatonin production is increased. Therefore, it is difficult to find resting EEG studies conducted during the day when daily activities are the most vigorous. This study aimed to confirm the stable pathological characteristics of chronic insomnia during the day by measuring resting EEG and electrocardiogram (ECG) signals, which are well-known indicators of hyperarousal. Then, EEG source analysis based on the frequency bands followed to determine which area of the brain is the source of ‘hyperaroused’ EEG signals.

## 2. Experimental Section

### 2.1. Participants

A total of 24 participants with chronic insomnia (21 females and 3 males, mean age = 22.58, SD = 2.18) and 22 normal sleepers (18 females and 4 males, mean age = 24.72, SD = 6.34) were recruited in Seoul, the Republic of Korea, through posters on social communities promoting the research. Insomnia patients (INS) who did not meet the criteria for the diagnosis of mental disorders other than insomnia through the Structured Clinical Interview for DSM-IV Axis I disorder [31], satisfied DSM-5 diagnostic criteria for insomnia disorder, and achieved scores of ≥5 on the Pittsburgh Sleep Quality Index (PSQI) [32] and ≥8 on the Insomnia Severity Index (ISI) [33] were enrolled in the study. They were excluded from participation if they had a diagnosed sleep disorder other than insomnia (e.g., obstructive sleep apnea syndrome, periodic limb movement disorder, and narcolepsy). In the case of positive scores in two or more categories of the Berlin Questionnaire (BQ) [34] with snoring more than four times per week and a body mass index (BMI) ≥30, insomniacs were considered as having high-risk sleep apnea and excluded from the study. Subjects who reported having an uncomfortable sensation associated with the urge to move their legs were considered as having restless legs syndrome and excluded. After receiving the previously provided sleep diary, we checked the pattern of sleep difficulty and confirmed the conditions that may affect the sleep-wake cycle such as shift work and jet lag to rule out circadian rhythm disorder. Normal sleepers (NSC) were excluded if the following conditions were met: (1) scores of <5 on the PSQI and <8 on the ISI; (2) did not meet the criteria for the diagnosis of psychiatric disorders by the SCID-I. Exclusion criteria for all participants were history of any general medical conditions and mental disorders, taking medications that could affect sleep within 1 month or having atypical sleep schedules (e.g., shiftwork, an overseas trip across time zones within 6 months), or confirmed cardiovascular disease. The study protocol was reviewed and approved by the Institutional Review Board of Duksung Women’s University (IRB no.20140627). All participants were right-handed. They were given information about the experiment and signed the consent form.

### 2.2. EEG Recording and Analysis

All participants were instructed to refrain from consuming caffeinated beverages or alcoholic beverages and smoking for 24 h before EEG/ECG measurements were performed. Physiological signals were collected between 12:00 and 4:00 p.m. The participants were seated and engaged in a resting state in a sound/light-shielded room to minimize external noise and confounding artifacts. The EEG data acquisition with the eyes open and the eyes closed condition each are supposed to be different states. For this reason, EEG recordings were conducted by switching between eyes open (EO) and eyes closed (EC) conditions and both 2 min conditions were measured twice each. The combination of EO and EC was presented in one of six orders randomly (OCOC; OOCC; OCCO; COOC; COCO; CCOO) [35,36,37].

EEG recordings and acquisitions were made using Brain Master Discovery 20 (BrainMaster Technologies, Inc., Bedford, OH, USA) with a 19-channel Electro-cap (Electro-Cap International, Inc., Bedford, OH, USA), based on the international 10–20 system of electrode placement (Fp1, Fp2, F7, F3, Fz, F4, F8, T3, C3, Cz, C4, T4, T5, P3, Pz, P4, T6, O1, O2). Linked-ears reference montage was used. EEG signals were sampled at 256 Hz per second and filtered using a 60Hz notch filter to remove artifacts from the signals. EEG data were analyzed using NeuroGuide 3.0.5 software (NG; Applied Neuroscience, Inc., Tampa, FL, USA). EEG recordings were visually and automatically examined to eliminate eye muscle movements and other artifacts. The threshold for acceptable data quality for each EEG data file was a minimum of 60 s of artifact-free records with >95% split-half reliability and >90% test-retest reliability. Accepted EEG data were subjected to spectral analyses using a fast Fourier transform algorithm (FFT). EEG power spectra were calculated across the following bands: delta (1–4 Hz), theta (4–8 Hz), alpha (8–12 Hz), beta (12–25 Hz), high beta (25–30 Hz), and gamma (30–40 Hz). Source analysis using the swLORETA method was performed to localize the brain area in the NG Deluxe 3.0.5 system, which uses the boundary element method (BEM) to compute the inverse solution and a real-MRI with 12,270 voxels. Using NeuroNavigator software, 20 Brodmann area (BA) regions of interest (ROIs) within default mode network (DMN) were selected from a total of 96 BAs for source localization analysis. ROIs within the DMN were as follows for both left (L) and right (R) sides: the prefrontal cortex (PFC, BA 10), orbital frontal cortex (BA 11), occipital cortex (OC, BA 19), posterior and cingulate and temporal gyrus (PCC/STC, BA 29), postcentral gyrus (PCG, BA 2), PCC and cuneus (PCC/Pcu, BA 30), medial temporal lobe and parahippocampal gyrus (MTL/PHG, BA 35), angular gyrus and inferior parietal lobe (AG/IPL, BA 39), inferior parietal lobe (IPL, BA 40), and supramarginal gyrus (SMG, BA 7) [38].

### 2.3. ECG Recording and Analysis

After EEG data acquisition, the raw ECG signal was obtained with the ProComp 5 Infiniti System (Thought Technology Ltd., Montreal, QC, Canada) at a sampling frequency of 2048 Hz using an ECG-Flex/Pro sensor with three electrodes attached to the chest. ECG data were collected for 5 min and transformed to heart rate variability (HRV) indices. The heart rate (HR) was acquired in beats per minute and HRV analysis was performed using a frequency domain method. The frequency bands considered were the low-frequency (LF; 0.05–0.15 Hz) and high-frequency (HF; 0.15–0.4 Hz) bands. The LF band reflects sympathetic activity whereas the HF band reflects parasympathetic nervous system activity. The ratio of high frequency to low frequency (LF/HF ratio) means the balance between sympathetic and parasympathetic nervous system activity.

### 2.4. Statistical Analysis

R 3.4.4 was used for statistical analysis. To estimate differences between groups, we used the linear model (LM) for clinical variables and the generalized linear mixed model (GLMM) for gamma distribution with log link for HRV and functional connectivity because of the non-normality of the distribution. As the Beck Depression Inventory (BDI) and the Beck Anxiety Inventory (BAI) scores were significantly higher in the insomnia group than in the control group, these two variables were used as covariates in the analysis. For multiple testing corrections, the Benjamini and Hochberg method was used to control the region-wise false discovery rate (FDR). The 19 sites were averaged into 3 regions: F (Fp1, Fp2, F7, F3, Fz, F4, F8), C (T3, C3, Cz, C4, T4), P (T5, P3, Pz, P4, T6, O1, O2).

## 3. Results

### 3.1. Participants

There were no significant differences in age, education level, or BMI between patients with INS and NSC (Table 1). As expected from the inclusion criteria, compared with NSC, INS had higher scores on the PSQI and ISI. The level of daytime sleepiness based on the Epworth Sleepiness Scale (ESS) [39] was statistically different between the two groups and indicated that INS complained more of daytime sleepiness. Self-report questionnaires confirmed that INS also showed significantly higher levels of arousal before sleep at night as measured by the Pre-Sleep Arousal Scale (PSAS) [40]. The total BDI and BAI scores of NSC were significantly higher than those of INS.

### 3.2. HRV of Insomniacs and Normal Sleepers

**Figure 1:**
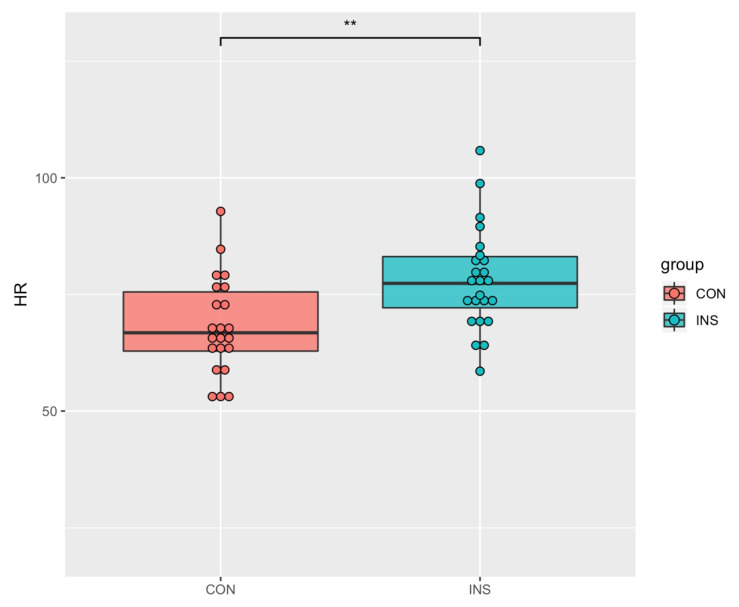
Significant differences between INS and NSC of heart rate. INS showed more increased heart rate than NSC in ECG analysis. ** *p* < 0.001.

A comparison of HRV indices showed statistically significant differences only in the HR between the two groups (Table 1, Figure 1). INS showed an increased HR in ECG analysis (INS = 78.17 (10.88), NSC = 68.24 (10.39), *p* < 0.050). The LF band and LF/HF ratio, which reflect the activation of sympathetic activity in the autonomic nervous system, were higher among INS; however, the result was not statistically significant. Analysis of all participants showed a positive correlation of HR with all psychological variables (Table 2). In addition, positive correlations were found between the LF band and PSQI (r = 0.282, *p* < 0.0001), ISI (r = 0.224, *p* < 0.0001), BDI (r = 0.263, *p* < 0.0001), and PSAS (r = 0.089; *p* < 0.050) and between the LF/HF ratio and PSQI (r = 0.111, *p* < 0.050) and BDI (r = 0.215; *p* < 0.0001). However, the HF band, which reflects the activity of the parasympathetic nervous system, was negatively correlated with the PSQI, ESS, PSAS, BDI, and BAI (r = −0.128, r = 0.139, −0.141, −0.263, and −0.160, respectively *p* < 0.0001).

### 3.3. EEG Activity

As shown in Table 3, with analysis under EO condition, the group effect of the delta (χ^2^ = 461.895, *p* < 0.0001), high beta (χ^2^ = 3.934, *p* < 0.050), and gamma (χ^2^ = 5.095, *p* < 0.050) bands were significant. The group-by-region interaction effect was significant at all frequency bands; post-hoc test results showed that INS had higher absolute power in the delta band (χ^2^ = 25.509, *p* < 0.0001) and theta band (χ^2^ = 10.979, *p* < 0.050) in the frontal region (frontal *P*_FDR_ < 0.0001) and the beta band (χ^2^ = 8.295, *p* < 0.050) in the central and parietal regions (central and parietal *P*_FDR_ < 0.050). Under EC condition, the group and group-by-region interaction effects were significant only in the high beta band (Table 4); post-hoc test results demonstrated that the absolute power in the high beta band of the INS was higher than that of the NSC in all regions (χ^2^ = 829.583, *p* < 0.0001, frontal, central, and parietal *P*_FDR_ < 0.050). 

### 3.4. Brain Source Localization of INS 

**Figure 2:**
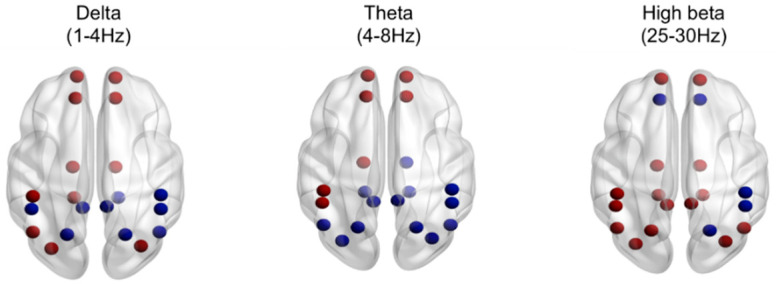
Source network analysis using 20 Brodmann area (BA) regions of interest (ROIs) within default mode network (DMN) during the eyes open condition in the delta (1–4 Hz), theta (4–8 Hz), and high beta (25–30 Hz) bands. Red dots indicate the higher frequency spectral power of insomnia patients compared with normal sleepers, whereas blue dots indicate the opposite. *p* < 0.050.

**Figure 3:**
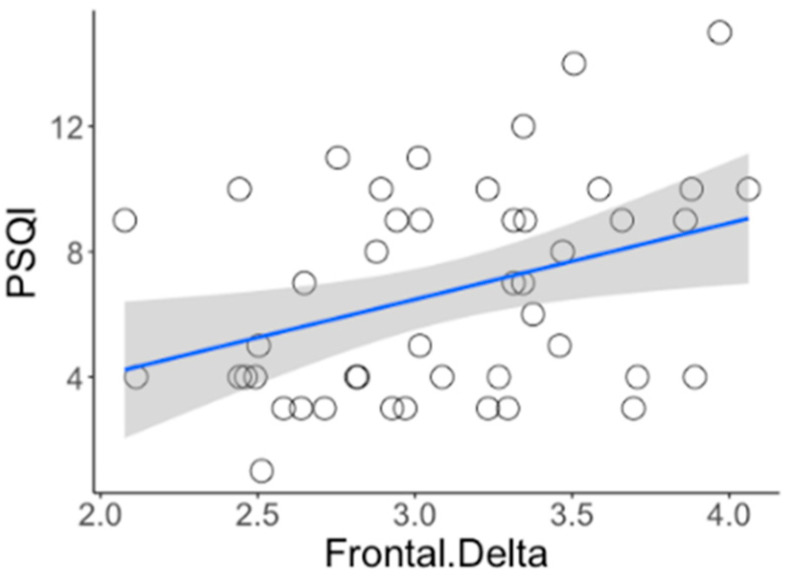
Correlation between the delta band in the frontal region and PSQI of all participants (rho = 0.376, *P*_FDR_ < 0.050).

The result of absolute power analysis showed statistical differences between INS and NSC in both the EO and EC conditions, and source level analysis was performed to identify the correct brain area. There were no significant differences under the EC condition. However, significant differences were observed under the EO condition. As shown in Figure 2, the delta and theta bands were predominant in the frontal lobe, whereas the high beta band was distributed throughout the entire brain area, including the prefrontal, medial, and occipital lobes. Specific regions with significant differences are shown in the Supplementary Materials. Additionally, the Spearman correlation analysis of all participants between the brain wave and self-report measures identified a positive correlation between the delta band in the frontal region and the PSQI (rho = 0.376, *P*_FDR_ < 0.050, Figure 3). 

## 4. Discussion

The purpose of the study was to confirm whether the hyperarousal state of individuals with chronic insomnia was persistent during the day. Patients satisfying the DSM-5 criteria for insomnia without other coexisting mental disorders underwent psychophysiological assessments such as EEG and ECG to identify pathological and neurophysiological factors among those with chronic insomnia. The finding showed that individuals with chronic insomnia were in a state of neurophysiological hyperarousal during the middle of the day.

Patients with chronic insomnia showed significantly higher slow-wave activity (delta and theta bands) in the frontal region and fast brain wave activity (high beta band) in the central and parietal regions during the EO condition. Moreover, these results were consistent with the finding of source network analysis: slow-wave activity was mainly increased in the frontal lobe whereas fast-wave activity was increased in broad areas of the prefrontal, central, parietal, and occipital lobes. The results of this study are an extension of previous findings, which showed high-frequency EEG activity was observed during sleep-onset and NREM sleep and in the morning after wakefulness [18,22,23,41,42,43,44,45]. All these results suggest hyperarousal persists during the day when individuals are active. Colombo et al. [30] obtained waking-state EEG data late in the evening, and the study showed reduced power in the upper alpha band under the EO condition and increased power in the beta and gamma band under the EC condition for patients with insomnia. In our study, we attempted to investigate the possibility that this state may be observed even earlier as it could be an all-day phenomenon.

The relationship between slow-wave and fast-wave activity is known as the cross-talk between the subcortical and cortical brain regions [46,47]. Increased slow-fast wave coupling is considered to reflect increased attentional control [48] and cortisol levels [49], and many studies using fMRI also found that chronic insomnia patients showed abnormal brain connectivity [50,51]. In this regard, this result may suggest that insomnia patients have higher cognitive activity and greater load on the brain by accepting excessive information from external stimuli. In contrast to the EO condition, only increased high beta band activity was observed in all regions during the EC condition. Increased fast wave activity is associated with attention-concentration processes such as inhibition control or integration of sensation and perception [52,53]. As most patients with insomnia have a hard time suppressing unwanted thoughts and worries [54,55], increased high-frequency activity may indicate the presence of these detrimental cognitive effects at the neurophysiological level.

The high-frequency activity was prevalent under the EC condition, and slow-fast frequency coupling was observed under the EO condition. The slightly different EEG results suggest that insomniacs may have slightly different difficulties under each condition. Under the EO condition, with increased perceptual load, insomniacs are in a high state of tension and anxiety and exhibit cortical activation; thus, they are always alert to small stimuli from outside and are easily aroused. In contrast, the EC condition can be interpreted as an attempt to rest by blocking the entry of external stimuli. However, similar to difficulties in sleeping at night, attempts to rest during the day may fail because of disruptive internal stimuli. In addition, the present HR measurement results are consistent with those of previous studies involving patients with chronic insomnia [9,56,57]. A comparison of insomniacs and normal sleepers revealed that the HR of those with insomnia was statistically higher. Furthermore, the more individuals perceive themselves as having poor sleep quality and severe insomnia symptoms at night, the higher the sympathetic nervous system activity (indicated by the LF band and LF/HF ratio). These findings suggest that daytime sympathetic nervous system activity may predict the severity of individual sleep problems.

Taken together, a daytime hyperarousal state may describe individuals who cannot sleep easily due to abnormal sleep-arousal regulation. This critical condition originates from daytime cortical alertness and extends to the pre-sleep state and sleep time. The brain of people with insomnia is generally hyperaroused, which makes it difficult for them to fall asleep. It can be inferred that individuals with chronic insomnia have difficulties in getting sufficient rest at any time and this vulnerability can be detected by a simple EEG test performed during the day.

The present study identified a hyperaroused EEG pattern during the day and suggested a more time-and-cost efficient measurement alternative to evaluate insomnia. In addition, our study provided important information on the electrode placement and frequency band applicable to intervention to control daytime hyperarousal such as neurofeedback training. It is very important to confirm the exact frequency band and the placement of the electrode that will be the target of neurofeedback training.

However, some limitations should be noted. First, it focused on individuals in their early 20s and most of the participants were women. Further studies are needed with subjects from various demographic backgrounds to allow for generalization to a wider population of patients with insomnia. Second, we selectively recruited insomnia disorder without comorbidities. It is unclear whether our findings will apply similarly to patients with insomnia with other mental disorders or medical problems. Therefore, future studies should use both objective and subjective information in the diagnosis of different types of sleep-related disorders. Nevertheless, to the best of our knowledge, this is the first attempt to identify the pathophysiological characteristics of the hyperarousal state by evaluating the psychophysiological characteristics of chronic insomnia during the day. This study could contribute to a better understanding of biological markers for patients with insomnia

## Figures and Tables

**Table 1:** Demographic and clinical characteristics of subjects (M, SD).

Variables	INS (*n* = 24)	NSC (*n* = 22)	t	*p*
Sex				
Male	3 (12.5%)	4 (18.18%)		
Female	21 (87.5%)	18 (81.81%)		
Age	22.58 (2.18)	24.72 (6.34)	−1.22	0.226
Education	15.33 (0.91)	15.68 (1.42)	−0.99	0.325
BMI	20.30 (2.04)	21.07 (2.38)	−1.18	0.243
PSQI	9.50 (2.16)	3.68 (0.99)	11.52	<0.0001 ***
ISI	14.13 (4.27)	2.18 (1.53)	12.38	<0.001 ***
ESS	8.50 (3.28)	6.32 (3.56)	2.16	0.036 *
PSAS	46.46 (9.28)	30.18 (8.27)	6.25	<0.001 ***
BDI	10.38 (6.27)	5.55 (4.48)	2.97	0.004 *
BAI	6.96 (3.99)	4.05 (3.74)	2.54	0.014 *
HR (beats/min)	78.17 (10.88)	68.24 (10.39)	3.15	0.002 *
HF (%)	40.68 (14.69)	42.28 (18.90)	−0.32	0.748
LF (%)	33.60 (11.25)	29.71 (10.48)	1.2	0.233
LF/HF ratio	1.39 (1.31)	1.22 (1.14)	0.48	0.626

Note. Data are given as mean (SD). BMI, body mass index; PSQI, Pittsburgh Sleep Quality Index; ISI, Insomnia Severity Index; ESS, Epworth Sleepiness Scale; PSAS, Pre-sleep Arousal Scale; BDI, Beck Depression Index; BAI, Beck Anxiety Index; HR, heart rate (heart rate per minute); HF, high frequency (0.15–0.4 Hz); LF, low frequency (0.04–0.15 Hz); LF/HF ration, ratio of high frequency to low frequency; INS, insomnia patients; NSC, normal sleeper controls. * *p* < 0.050, *** *p* < 0.0001.

**Table 2:** Correlation (including 95% confidence intervals (CI)) between HRV and self-report measures of all subjects.

	HR	HF (%)	LF (%)	LF/HF Ratio
PSQI	0.270 ***	−0.128 ***	0.282 ***	0.111 *
	(0.007 to 0.556)	(−0.391 to 0.17)	(0.05 to 0.48)	(−0.182 to 0.388)
ISI	0.319 ***	−0.033	0.224 ***	0.003
	(0.085 to 0.554)	(−0.279 to −0.139)	(−0.020 to 0.446)	(−0.248 to 0.268)
ESS	0.082 *	−0.139 ***	0.047	0.046
	(0.001 to 0.138)	(−0.496 to 0.191)	(−0.247 to 0.334)	(−0.279 to 0.371)
PSAS	0.292 ***	−0.141 ***	0.089 *	0.019
	(0.042 to 0.524)	(−0.372 to 0.126)	(−0.172 to 0.336)	(−0.262 to 0.288)
BDI	0.235 ***	−0.263 ***	0.263 ***	0.215 ***
	(0.049 to 0.513)	(−0.487 to −0.015)	(−0.008 to 0.503)	(0.006 to 0.404)
BAI	0.078 *	−0.160 ***	0.026	0.026
	(−0.193 to 0.349)	(−0.369 to 0.092)	(−0.247 to 0.288)	(0.211 to 0.273)

Note: PSQI, Pittsburgh Sleep Quality Index; ISI, Insomnia Severity Index; ESS, Epworth Sleepiness Scale; PSAS, Pre-Sleep Arousal Scale; BDI, Beck Depression Index; BAI, Beck Anxiety Index; HR, heart rate (heart rate per minute); HF, high frequency (0.15–0.4 Hz); LF, low frequency (0.04–0.15 Hz); LF/HF ration, ratio of high frequency to low frequency; INS, insomnia patients; NSC, normal sleeper controls. * *p*< 0.050, *** *p*< 0.0001. 95% CI was calculated with bootstrap.

**Table 3:** Absolute power during the eyes open condition.

	INS (*n* = 24)	NSC (*n* = 22)	Group χ^2^	Group*Region χ^2^	*P*_FDR_
Delta			461.895 ***	25.509 ***	
F	27.17 (18.23)	21.52 (15.17)			0.001 **
C	13.98 (10.41)	14.45 (11.11)			0.559
P	13.18 (5.81)	13.93 (6.85)			0.774
Theta			0.159	10.979 *	
F	10.48 (5.35)	8.43 (3.91)			<0.0001 ***
C	8.31 (4.60)	7.85 (4.80)			0.698
P	8.45 (3.95)	8.28 (4.39)			0.491
Alpha			0.435	6.929 *	
F	7.19 (5.68)	8.74 (7.32)			
C	8.85 (9.44)	11.23 (10.62)			
P	15.61 (19.5)	22.13 (24.88)			
Beta			0.991	7.300 *	
F	7.14 (4.90)	7.49 (8.71)			0.950
C	8.23 (6.48)	7.24 (4.44)			0.375
P	7.83 (6.35)	8.77 (6.41)			0.950
High beta			3.934 *	8.295 *	
F	1.98 (2.42)	1.89 (3.78)			
C	1.92 (2.36)	1.17 (1.06)			
P	1.10 (0.61)	0.90 (0.49)			
Gamma			5.095 *	13.190 **	
F	2.72 (3.73)	2.7 (5.36)			0.057
C	3.2 (5.33)	1.86 (2.46)			0.886
P	1.26 (0.69)	1.21 (0.99)			0.886

Note: group difference between INS and NSC during the eyes-closed (EC) condition. F, frontal region; C, central region; P, parietal region. * *p* < 0.050, ** *p* < 0.001, *** *p* < 0.0001.

**Table 4:** Absolute power during the eyes closed condition.

	INS (*n* = 24)	NSC (*n* = 22)	Group χ^2^	Group*Region χ^2^	*P*_FDR_
Delta			0.003	5.043	
F	22.02 (13.74)	20.24 (12.74)			
C	14.42 (8.76)	14.38 (8.61)			
P	15.62 (7.02)	15.62 (8.27)			
Theta			0.518	2.515	
F	12.18 (5.50)	10.46 (4.91)			
C	10.83 (5.77)	9.91 (5.85)			
P	12.96 (6.01)	11.82 (5.65)			
Alpha			0.082	1.042	
F	17.46 (16.73)	17.01 (12.67)			
C	21.63 (23.63)	21.18 (20.54)			
P	50.07 (52.98)	48.25 (36.31)			
Beta			0.037	1.579	
F	7.06 (3.17)	6.65 (3.00)			
C	8.71 (4.77)	8.24 (4.56)			
P	13.29 (7.99)	13.29 (7.7)			
High beta			2019.460 ***	829.583 ***	
F	1.27 (0.68)	1.10 (0.67)			0.011 *
C	1.39 (1.04)	1.04 (0.62)			0.003 *
P	1.27 (0.64)	1.15 (0.60)			0.047 *
Gamma			8.892	1.434	
F	1.52 (0.93)	1.45 (0.98)			
C	1.69 (1.66)	1.44 (1.46)			
P	1.31 (0.57)	1.39 (1.10)			

Note: group difference between INS and NSC during the eyes-closed (EC) condition. F, frontal region; C, central region; P, parietal region. * *p* < 0.050, *** *p* < 0.0001.

## Supplementary Materials

The following are available online at https://www.mdpi.com/2077-0383/9/11/3425/s1, Table S1: Source network analysis during eyes closed condition, Table S2: Source network analysis during eyes open condition.
