# Supplementary material for: Daytime Neurophysiological Hyperarousal in Chronic Insomnia: A Study of qEEG
Source: J Clin Med. 2020 Oct 26;9(11):3425. doi: 10.3390/jcm9113425 (PMC7694040; doi:10.3390/jcm9113425)
Supplement: Supplementary file 1 [file jcm-09-03425-s001.pdf]

Supplementary material

S1. Source network analysis during eyes closed condition.

Region		Delta	*P*FDR	Theta	PFDR	High beta	*P*FDR
10L	Pre-frontal cortex	0.862	NS	1.003	NS	1.122	NS
10R		1.141		1.407		1.301	
11L	Orbital frontal	0.859		1.111		1.346	
11R		1.350		1.322		1.762	
19L	Occipital cortex	0.164		0.169		1.300	
19R		0.606		0.085		0.581	
29L	Posterior	0.176		0.048		1.161	
29R	cingulate&superior transverse temporal gyrus	0.158		-0.066		0.748	
2L	Post central gyrus	-0.539		0.435		1.676	
2R		-0.644		0.178		1.638	
30L	Posterior	0.778		0.293		1.029	
30R	cingulate&cuneus	0.680		0.093		0.520	
35L	Medial temporal	0.710		0.594		1.412	
35R	lobe&para hippocampal gyrus	1.037		0.421		1.061	
39L	Angular	0.336		0.510		2.260	
39R	gyrus&inferior parietal lobe	0.334		0.174		0.804	
40L	Inferior parietal	-0.178		0.576		2.199	
40R	lobe angular gyrus	-0.502		0.168		1.530	
7L	Supramarginal	-1.331		-0.313		1.347	
7R	gyrus	-1.045		-0.292		1.165	

Note. L, Left; R, right; NS, Not Significant (p<.050)

S2. Source network analysis during eyes open condition.

Region		Delta	PFDR	Theta	PFDR	High beta	PFDR
10L	Pre-frontal cortex	8.294	0.000	6.727	0.000	-2.447	0.018
10R		10.526	0.000	6.923	0.000	-3.112	0.003
11L	Orbital frontal	10.098	0.000	6.516	0.000	-0.227	NS
11R		10.986	0.000	6.906	0.000	-1.713	NS
19L	Occipital cortex	3.916	0.000	0.959	NS	7.937	0.000
19R		2.878	0.005	-1.099	NS	5.063	0.000
29L	Posterior	-0.695	NS	-0.812	NS	8.379	0.000
29R	cingulate&superior	-3.088	0.003	-2.455	0.026	5.568	0.000

	transverse temporal gyrus						
2L	Post central gyrus	2.240	0.032	3.529	0.001	9.018	0.000
2R		-2.954	0.005	-2.099	NS	-2.518	0.016
30L	Posterior	4.874	0.000	1.329	NS	11.179	0.000
30R	cingulate&cuneus	1.237	NS	-0.886	NS	6.818	0.000
35L	Medial temporal	6.652	0.000	3.486	0.001	12.964	0.000
35R	lobe&para hippocampal gyrus	4.632	0.000	1.596	NS	8.166	0.000
39L	Angular	3.328	0.002	2.164	NS	9.793	0.000
39R	gyrus&inferior parietal lobe	-0.184	NS	-1.973	NS	6.133	0.000
40L	Inferior parietal	1.489	NS	2.847	0.010	10.596	0.000
40R	lobe angular gyrus	-3.848	0.000	-2.836	0.010	0.590	NS
7L	Supramarginal	-4.355	0.000	-2.466	0.026	7.272	0.000
7R	gyrus	-6.908	0.000	-5.243	0.000	1.572	NS

Note. L, Left; R, right; NS, Not Significant (p<.050)
